# Novel synthetic analogues of avian β-defensin-12: the role of charge, hydrophobicity, and disulfide bridges in biological functions

**DOI:** 10.1186/s12866-017-0959-9

**Published:** 2017-02-23

**Authors:** Ming Yang, Chunye Zhang, Michael Z. Zhang, Shuping Zhang

**Affiliations:** 1 Department of Veterinary Pathobiology, College of Veterinary Medicine, University of Missouri, Columbia, MO 65211 USA; 2 Department of Biomedical Science, College of Veterinary Medicine, University of Missouri, Columbia, MO 65211 USA; 3 Veterinary Medical Diagnostic Laboratory, College of Veterinary Medicine, University of Missouri, Columbia, MO 65211 USA

**Keywords:** Avian β-defensin analogues, Antimicrobial activity, Chemotactic activity, Net positive charge, Hydrophobicity, Disulfide bridges

## Abstract

**Background:**

Avian β-defensins (AvBD) possess broad-spectrum antimicrobial, LPS neutralizing and chemotactic properties. AvBD-12 is a chemoattractant for avian immune cells and mammalian dendritic cells (JAWSII) — a unique feature that is relevant to the applications of AvBDs as chemotherapeutic agents in mammalian hosts. To identify the structural components essential to various biological functions, we have designed and evaluated seven AvBD analogues.

**Results:**

In the first group of analogues, the three conserved disulfide bridges were eliminated by replacing cysteines with alanine and serine residues, peptide hydrophobicity and charge were increased by changing negatively charged amino acid residues to hydrophobic (AvBD-12A1) or positively charged residues (AvBD-12A2 and AvBD-12A3). All three analogues in this group showed improved antimicrobial activity, though AvBD-12A3, with a net positive charge of +9, hydrophobicity of 40% and a predicted CCR2 binding domain, was the most potent antimicrobial peptide. AvBD-12A3 also retained more than 50% of wild type chemotactic activity. In the second group of analogues (AvBD-12A4 to AvBD-12A6), one to three disulfide bridges were removed via substitution of cysteines with isosteric amino acids. Their antimicrobial activity was compromised and chemotactic activity abolished. The third type of analogue was a hybrid that had the backbone of AvBD-12 and positively charged amino acid residues AvBD-6. The antimicrobial and chemotactic activities of the hybrid resembled that of AvBD-6 and AvBD-12, respectively.

**Conclusions:**

While the net positive charge and charge distribution have a dominating effect on the antimicrobial potency of AvBDs, the three conserved disulfide bridges are essential to the chemotactic property and the maximum antimicrobial activity. Analogue AvBD-12A3 with a high net positive charge, a moderate degree of hydrophobicity and a CCR2-binding domain can serve as a template for the design of novel antimicrobial peptides with chemotactic property and salt resistance.

**Electronic supplementary material:**

The online version of this article (doi:10.1186/s12866-017-0959-9) contains supplementary material, which is available to authorized users.

## Background

Rapid emergence of antimicrobial resistance poses a major global threat to public health and the economy [1, 2]. Excessive use and misuse of antibiotics in medicine and food production contribute to the rise of drug resistant pathogens [3, 4]. Control and prevention of antibiotic resistance call for holistic strategies including judicious use of antimicrobials, effective diagnostic tools, and novel therapeutic agents that are less likely to trigger resistance [5].

Beta-defensins are small, cationic, antimicrobial peptides found in different living organisms [6–9]. These endogenous peptides constitute the first line of innate defense against pathogenic bacteria, fungi, and viruses [10, 11]. Beta-defensins possess various biological properties, including broad-spectrum microbicidal activity, neutralization of LPS, activation of macrophages and dendritic cells, chemoattraction of dendritic cells, monocytes, and T-lymphocytes to the site of infection [7, 10, 12]. The microbicidal activity of defensins is achieved mainly through initial electrostatic attraction between positively charged amino acid residues of the peptides and negatively charged microbial surface components and subsequent microbial membrane damage which is followed by interacting with intracellular targets [13–15]. The chemotactic function of β-defensins is mediated by CC-chemokine receptors, such as CCR2 and CCR6 [16–18]. These natural antimicrobial peptides with complex mechanisms of action represent potentially a novel class of antimicrobials [13, 19]. However, several challenges must be addressed in order to develop β-defensins for therapeutic use which include retaining the biological activities under physiological conditions and the ease of production and purification of recombinant or synthetic peptides [20, 21].

Most defensin peptides characterized to date have a net positive charge, ranging from +2 to +9, and hydrophobicity of approximately 30-50% [15]. It has been reported that the three conserved disulfide bridges are required for the chemotactic function, but not the antimicrobial activity [17, 22–25]. Data from our laboratory showed that reduced (or linear form) AvBDs are fully active against bacterial pathogens whereas AvBDs without correctly folded disulfide bridges are not [21, 26]. To further understand the structural and functional characteristics of AvBDs, seven analogues were designed by replacing the negatively charged residues (D and E) and/or cysteines (C) with either positively charged residues (H, K, and R), hydrophobic residues (A, I, L, and V) or isosteric amino acids (Abu). The antimicrobial and chemotactic activities and salt-resistance of the analogues as well as their wild type parent peptides have been evaluated.

## Methods

### Design and synthesis of peptides

The three dimensional structures of AvBDs were predicted by using the I-TASSER program (http://zhanglab.ccmb.med.umich.edu/I-TASSER). The distribution of positively charged amino acids were evaluated using PyMOL (https://www.pymol.org/). Group 1 analogues, including AvBD-12A1, A2 and A3 were linear peptides, in which the six cysteines (C^1^C^2^C^3^C^4^C^5^C^6^ or C5C12C17C27C34C35) were replaced with structurally similar amino acid residues (alanine and serine) as follows: AvBD-12A1: A5A12A17A27A34A35, AvBD-12A2: S5S12S17A27A34A35, and AvBD-12A3: A5A12A17S27S34S35. Additional modifications were introduced to these analogues to alter peptide hydrophobicity and charge. In AvBD-12A1, the four negatively charged amino acid residues (D3D8E21E29) were replaced with one polar and three hydrophobic amino acid residues (H3V8L21I29). In AvBD-12A2 and AvBD-12A3, D3D8E21E29 were substituted with positively charged residues R3K8K21R29. Group 2 analogues, including AvBD-12A4, AvBD-12A5 and AvBD-12A6 had reduced number of disulfide bridges without any additional modifications. To remove disulfide bridges, the cysteine residues (C^1^C^2^C^3^C^4^C^5^C^6^ or C5C12C17C27C34C35) of AvBD-12 were replaced with isosteric α-aminobutyric acids (Abu, U) to create AvBD-12A4 (U5C12C17C27U34C35), A5 (U5U12C17U27U34C35), and A6 (U5U12U17U27U34U35). Group 3 included a single hybrid peptide, namely AvBD-12/6. This analogue was designed using the backbone of AvBD-12, in which the negatively charged amino acid residues (D3D8E21E29) of AvBD-12 were replaced with amino acids (H3Q8Y21S29) of AvBD-6 at the corresponding positions. The hydrophobicity and charge of AvBD analogues at neutral pH were calculated using Peptide 2.0 (http://peptide2.com) and Peptide property calculator (PepCalc.com), respectively.

All peptides were custom synthesized using the standard solid phase 9-fluorenylmethoxycarbonyl (Fmoc) method and purified by reverse phase high performance liquid chromatography (RP-HPLC) (Lifetein, Hillsborough, NJ). The analogues AvBD-12A4, −A5, AvBD-12/6, AvBD-6 and AvBD-12 with varying numbers of cysteine residues were subjected to oxidative folding as described previously [17]. Electrospray ionization mass spectrometry (ESI-MS) was performed to confirm the correct formation of disulfide bridges between Cys^1^-Cys^5^, Cys^2^-Cys^4^ and Cys^3^-Cys^6^. The purity of the synthetic AvBD analogues was over 98.5% as verified by liquid chromatography-mass spectrometry (LC-MS) (Lifetein, Hillsborough, NJ).

### Antimicrobial activity of AvBD analogues

*Escherichia coli* (ATCC 25922), *Pseudomonas aeruginosa (*ATCC 27853), *Salmonella enteric* serovar Typhimurium (ATCC 14028) and *Staphylococcus aureus* (ATCC 29213) were used to assess the antimicrobial activity of AvBD analogues. All bacterial strains were grown on Luria-Bertani (LB) agar plates or Trypticase Soy Agar with 5% Sheep Blood (TSA, Thermo Fisher Scientific) plates at 37 °C. Antimicrobial activity was determined by a colony counting assay [21, 26]. In brief, bacteria were resuspended in 100-fold diluted Mueller Hinton II broth with 5 mM NaCl (minimal growth medium) to obtain a final bacterial concentration of 2 *×* 10^5^ CFU/ml. Twenty-five microliters of bacterial suspension and 25 μl of AvBD analogue solution were mixed in the wells of a 96-well polypropylene microtiter plate (Nunc™, Thermo Fisher Scientific). The final peptide concentrations were 2, 4, 8, 16, 32, 64 and 128 μg/ml. The minimal growth medium without AvBD peptide was included as a negative control. The bacterial-peptide mixtures were incubated at 37 °C for 2 h, ten-fold serially diluted and plated on LB agar plates. The numbers of bacteria colonies were enumerated after 16 h of incubation at 37 °C. Antimicrobial activity was expressed as percent of killing using the following formula: (CFU_control_ - CFU_treated_) / CFU_control_ × 100%. To assess the resistance of AvBD analogues to sodium chloride (NaCl), antimicrobial assays were carried out in the presence of 5 mM, 50 mM, 100 mM or 150 mM NaCl. All assays were performed in triplicate.

### Minimum inhibitory concentrations of AvBD analogues

The minimum inhibitory concentrations (MIC) of AvBDs were determined according to the guidelines of the Clinical and Laboratory Standards Institute (CLSI) [27, 28]. The Muller Hinton II broth used in standard MIC assays contained 20–25 mg/L of calcium and 10–12.5 mg/L of magnesium. Modified MICs of AvBDs were also determined under a low-salt and low-nutrient condition by using 100× diluted Muller Hinton II broth containing 0.2 mg/L of calcium, 0.1 mg/L magnesium and 5 mM (292 mg/L) NaCl. MIC obtained under the low-salt and low-nutrient condition was referred to as MIC-ls. AvBD peptides were two-fold serially diluted (2 to 256 μg/ml) in a 96-well microtiter plate. An equal volume (μl) of bacterial suspension was added to each well of the plate. The final bacterial concentration in the wells was 5 × 10^5^ CFU/ml. The plate was incubated at 37 °C for 24 h and the lowest concentration that completely prevented visible bacteria growth was recorded. To complement MIC-ls assays, the minimum bactericidal concentrations (MBC) were evaluated by sub-culturing the contents of the first two clear wells obtained in the MIC-ls assay onto LB agar plates. All assays were conducted in triplicate. The lowest peptide concentration inhibiting more than 99% of bacterial growth was defined as MBC-ls. AvBD analogues were regarded as bactericidal if the MBC was no more than four times the MIC [29].

### Cell cytotoxicity assay

Chicken macrophage cell lines MQ-NCSU and HD11 were maintained in RPMI-1640 media supplemented with 10% fetal bovine serum (FBS), 2% chicken serum, 100 U/ml penicillin and 100 μg/ml streptomycin (Sigma-Aldrich) at 37 °C in humidified air with 5% CO_2_. CHO-K1 cells were cultured in the same media without 2% chicken serum. For CCR2-transfected CHO-K1 cells, the medium was supplemented with 500 μg/ml G418 was added (Sigma-Aldrich) [26]. Murine immature dendritic cell line JAWSII (ATCC CRL-11904™) was cultured in Alpha minimum essential medium containing 4 mM L-glutamine, 1 mM sodium pyruvate, 5 ng/ml murine Granulocyte macrophage colony-stimulating factor (GM-CSF), 20% FBS, 100 U/ml penicillin and 100 μg/ml streptomycin at 37 °C in humidified air with 5% CO_2_. The cell cytotoxicity was determined using the MTT (3-(4, 5-dimethylthiazol-2-yl)-2, 5-diphenyltetrazolium bromide metabolic activity assay according to the manufacturer’s instruction (Thermo Fisher Scientific). Briefly, cells (5 × 10^3^ cells/well) were seeded in 96-well tissue culture plates, incubated overnight and treated with AvBDs at concentrations of 4, 16, 64 and 256 μg/ml for 4, 12, 24 and 48 h at 37 °C. After treatment, 20 μl of 12 mM MTT solution was added to each well. The plate was incubated for 4 h and read at 540 nm. Viability was expressed as percentage of viable cells relative to the untreated control. The experiments were performed in triplicate.

### Chemotaxis assay

Migration of JAWSII and CCR2-CHO-K1 cells in response to AvBD-12 analogues was determined using a 48-well microchemotaxis chamber technique as previously described [30]. Chemotaxis buffer (Minimum Essential Medium containing 0.1% BSA, 100U/ml penicillin, and 100 μg/ml streptomycin) and bacterial peptide N-Formyl-methionyl-leucyl-phenylalanine (fMLF, Sigma-Aldrich) were included as negative and positive controls, respectively. The results were presented as chemotactic index (C.I.). C.I. = number of migrated cells induced by AvBDs / number of migrated cells induced by chemotactic buffer. The assay was repeated five times.

### Scanning electron microscopy (SEM)

SEM was performed according to the procedure described by Cobo *et al*. [31]. *S.* Typhimurium was cultured in Mueller-Hinton (MH) broth to mid-log phase and harvested by centrifugation at 5000 *g* for 10 min. Cell pellets were washed twice with 10 mM PBS and resuspended at a final number of 10^8^ CFU. The cell suspension was incubated with 1 × MIC-ls of AvBD-12A3, AvBD-12/6, wild-type AvBD-6 and AvBD-12 at 37 °C for 30 min. Bacterial pellets were fixed in 500 ml of 2.5% (v/v) glutaraldehyde in 0.2 M cacodylate buffer at 4 °C overnight, washed twice with 0.2 M cacodylate buffer and dehydrated through ethanol gradient (30%, 50%, 70%, 90%, 100% and again 100%) for 15 min in each gradient. The samples were transferred into a mixture (1:1, v/v) of ethanol and tertiary butanol and then pure tertiary butanol for 20 min each. After gold coating, the specimens were observed using a scanning electron microscope (Hitachi S-4700, Japan).

### Statistical analysis

Data were presented as the means ± standard deviation (SD). Differences between groups were analyzed using the one-way analysis of variance (ANOVA) followed by Duncan’s test for multiple comparisons using software SPSS version 19.0 (IBM Corp., Armonk, NY). Differences at *p* < 0.05 level were considered statistically significant, and at *p* < 0.01 level were considered extremely significant.

## Results

### Structural characteristics of AvBD-6 and AvBD-12 and their analogues

Substitutions of C^1^C^2^C^3^C^4^C^5^C^6^ (or C5C12C17C27C34C35) by A5A12A17A27A34A35 (AvBD-12A1) not only eliminated the three disulfide bridges, but also elevated the hydrophobicity from 33% (AvBD-12) to 47%. Additional H3V8L21I29 for D3D8E21E29 substitutions further increased the hydrophobicity to 53% and the net positive charge from +1 (AvBD-12) to +5 (AvBD-12A1). Changes from C^1^C^2^C^3^C^4^C^5^C^6^ to S5S12S17A27A34A35 (AvBD-12A2) or to A5A12A17S27S34S35 (AvBD-12A3) and changes from D3D8E21E29 to R3K8K21R29 (AvBD-12A2 and A3) eliminated all three disulfide bridges and increased the peptide hydrophobicity from 33% to 40% and the net positive charge from +1 to +9. AvBD-12A4, A5, and A6 respectively lost disulfide bridge(s) C^1–5^; C^1–5^ and C^2–4^; C^1–5^, C^2–4^, and C^3–6^. C to U changes did not affect the peptide hydrophobicity and charge. The hybrid AvBD-12/6 with the backbone of AvBD-12 and the positively charged amino acids (H3Q8Y21S29) of AvBD-6 retained parent peptides’ hydrophobicity (33%) and an intermediate charge (+5), compared to AvBD-6 (+7) and AvBD-12 (+1). The sequence, charge, hydrophobicity and number of disulfide bridges of analogues and wild-type AvBD-12 and AvBD-6 were listed in Table 1.

**Table 1:**
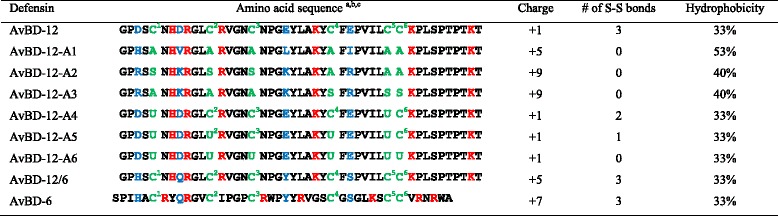
Amino acid sequences of avian β-defensin-12 (AvBD-12) analogues

^a^Disulfide bridges (S-S) between C^1–5^, C^2–4^, C^3–6^

^b^U: α-aminobutyric acid

^c^Acidic amino acids, basic amino acids, and cysteines are highlighted in blue, red, and green color, respectively. C^1^C^2^C^3^C^4^C^5^C^6^ or C5 C12 C17 C27 C34 C35

**Fig. 1:**
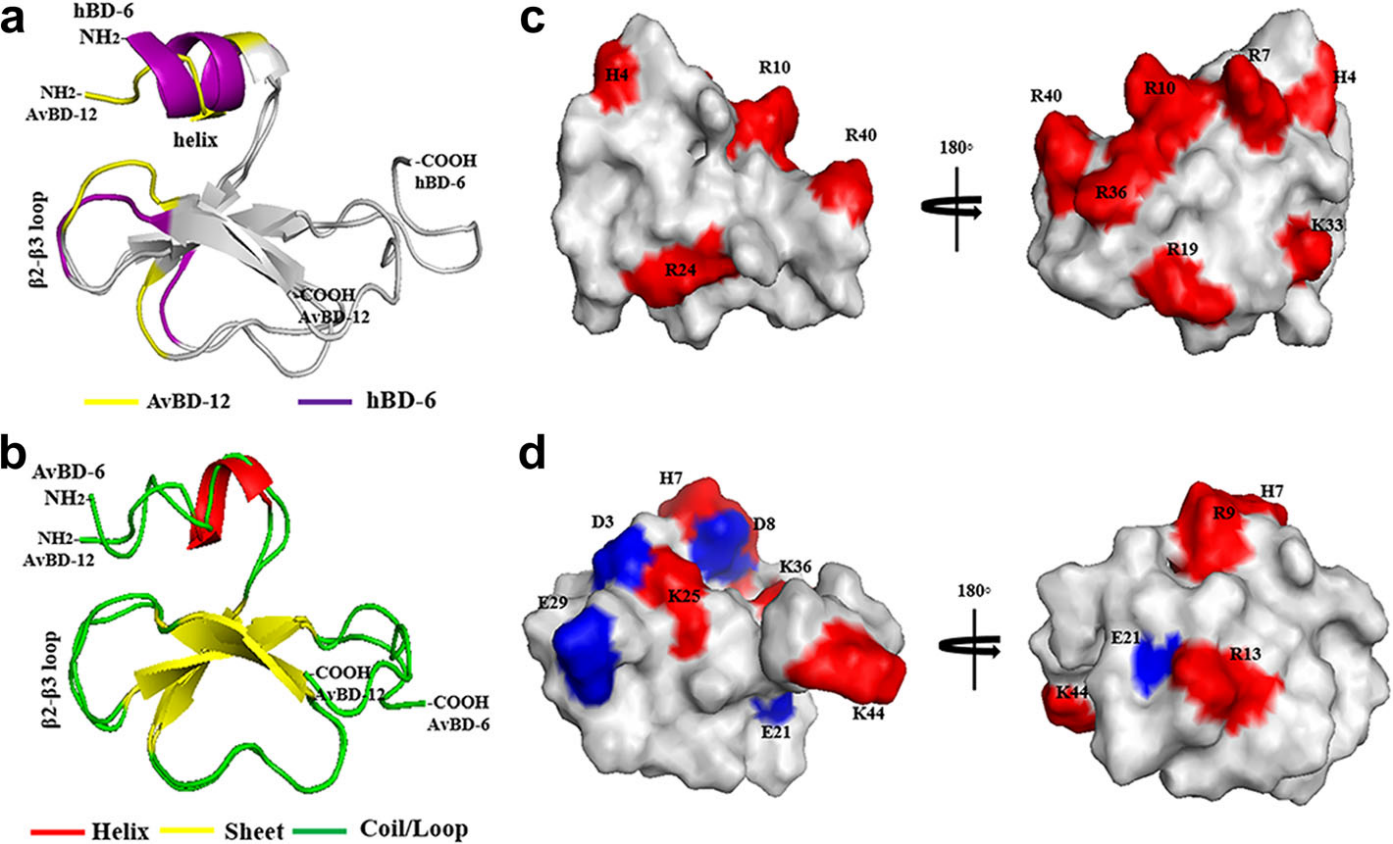
The predicted three dimensional structures of AvBD-6 and AvBD-12. I-TASSER online service program was used to predict peptide structures. **a** Superimposition of the three dimensional structures of AvBD-12 and hBD-6. The CCR2 binding surface of hBD6 is highlighted in purple and the corresponding region in AvBD-12 is highlighted in yellow. **b** Superimposition of AvBD-12 and AvBD-6. **c** Distribution of positively and negatively charged amino acid residues in AvBD-12. **d** Distribution of positively charged amino acids in AvBD-6. Basic and acidic amino acids are highlighted in red and blue, respectively

**Fig. 2:**
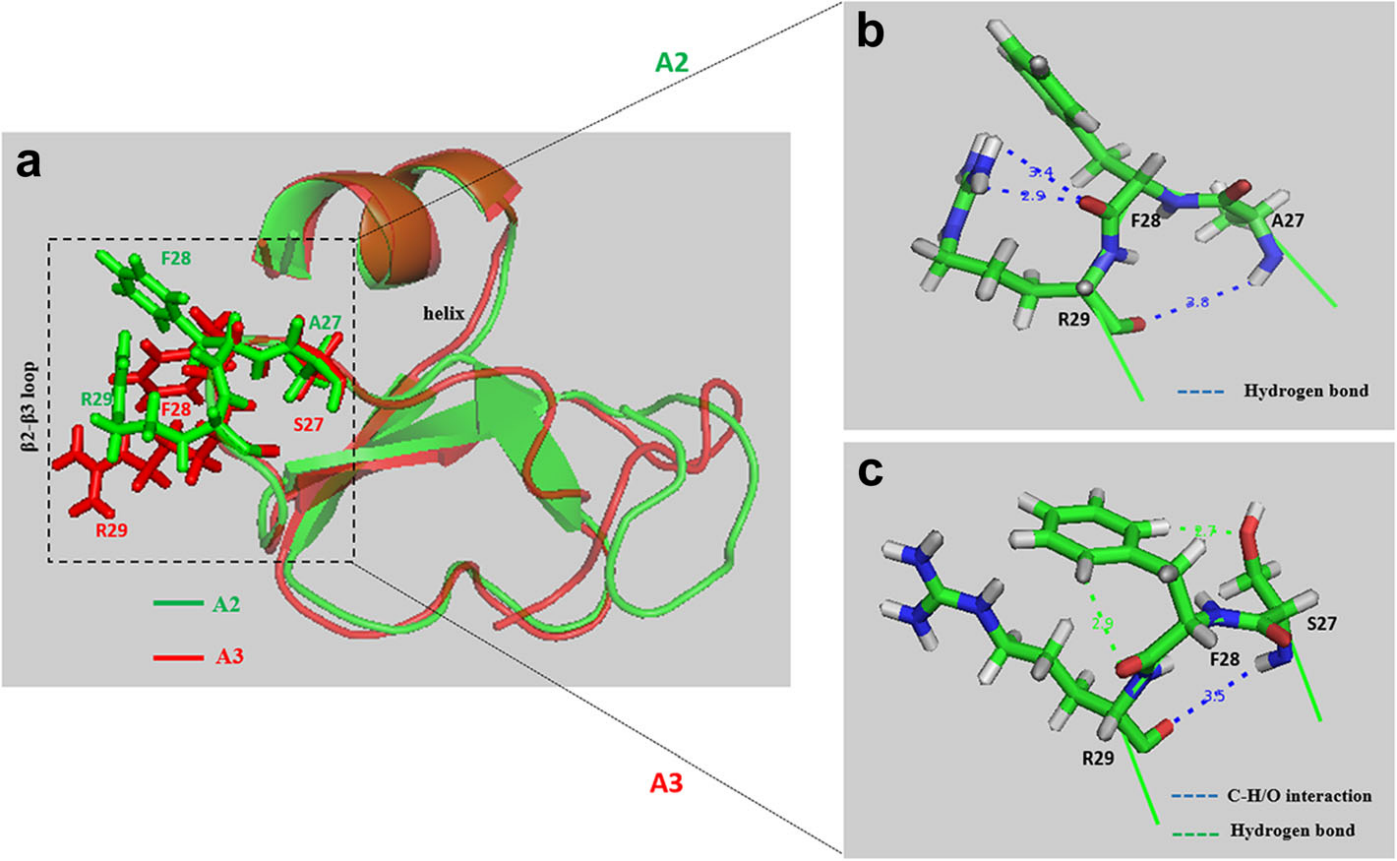
The predicted β2-β3 loop in AvBD-12A2 and AvBD-12A3. **a** Superimposition of AvBD-12A2 (green) and AvBD-12A3 (red) revealing the structural differences in the β2-β3 loop, a component of CCR2 binding domain. **b** Enlarged review of the β2-β3 loop in AvBD-12A2. The hydrogen bond between the -C = O group of F28 main chain and the -NH group of R29 side chain causes the arginine residue to fold back. **c** Enlarged review of the β2-β3 loop in AvBD-12A3. The CHO interactions between S27 -OH groups and the -CH groups on the aromatic ring of F28 result in an outward protrusion of R29 and a parallel twist of F28 aromatic ring. Distance: Å

Superimposition of the predicted three dimensional structures of AvBD-12 and the structure of human β-defensin 6 (hBD6) revealed a similar N-terminal α-helix and an adjacent β2-β3 loop, in addition to the conserved internal β-sheet domains (Fig. 1a). The α-helix and β2-β3 loop have been identified by NMR spectroscopy as a contiguous binding surface for human CCR2 [32]. A comparison of the predicted three dimensional structures of AvBD-6 and AvBD-12 showed only the β2-β3 loop in AvBD-6. AvBD-6 had an N-terminal coil turn instead of an α-helix. Peptide charge distribution analysis indicated that positively charged amino acid residues (H4R7R10R38R40) in the N- and C-termini of AvBD-6 formed a cluster whereas positively charged residues (H7R9K36) of AvBD-12 were separated by negatively charged residues (Fig. 1d). The N-terminal α-helix and the β2-β3 loop were also seen in the predicted structures of AvBD-12A2 and AvBD-12A3. However, differences in the β2-β3 loop between AvBD-12A2 and A3 were identified (Fig. 2a). In AvBD-12A2, the -C = O group in the main chain of F28 formed a hydrogen bond with the -NH group in the side chain of R29, resulting in the fold-back of R29 sidechain (Fig. 2b). In AvBD-12A3, there was no hydrogen bond formation between R29 and F28. Instead, there are C-H/O interactions between the -CH groups in the aromatic π ring of F28 and the -C = O group of F28 as well as the -CH group in the side chain of S27, similar to what was reported previously [33]. Consequently, the side chain of R29 protruded towards the surface of the peptide and the aromatic ring of F28 in AvBD-12A3 turned in parallel with R29 side chain (Fig. 2c).

### Antimicrobial activity

**Fig. 3:**
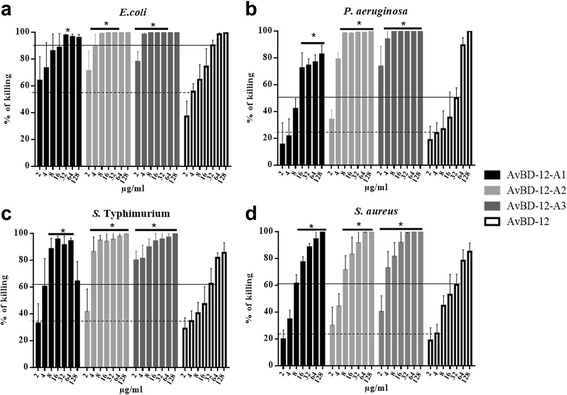
Antimicrobial activity of group 1 analogues. Bacteria (10^5^ CFU/ml) were incubated with peptides at various concentrations, ranging from 2 to 128 μg/ml at 37 °C for 2 h. Antimicrobial activity was presented as percent of killing compared to non-AvBD treated control. Antimicrobial activity of analogues against *E. coli* (**a**), *P. aeruginosa* (**b**), *S.* Typhimurium (**c**) and *S. aureus* (**d**). Data are means ± SD (*n =* 3). Statistical analysis was performed using one-way analysis of variance (ANOVA) followed by Duncan’s test for multiple comparisons using software SPSS version 19.0 (IBM Corp., Armonk, NY). Asterisk indicates statistically significant difference between the analogues and AvBD-12 at the same concentrations (*p < 0.05). Solid line: average killing percent of 32 μg/ml of wild-type AvBD-12. Dash line: average killing percent of 4 μg/ml of wild-type AvBD-12

Group 1 analogues with increased net positive charge and hydrophobicity were significantly more effective (p < 0.05) than the parent peptide AvBD-12 in killing *E. coli*, *S*. Typhimurium, *P. aeruginosa*, and *S. aureus* (Fig. 3). At 16 μg/ml, AvBD-12, AvBD-12A1, AvBD-12A2 and AvBD-12A3 killed 74.4%, 88.6%, 100%, 100% of *E. coli*; 47.3%, 95.6%, 93.9%, and 93.9% *S.* Typhimurium; 35.5%, 72.3%, 98%, and 100% of *P. aeruginosa*; and 52.9%, 77.2%, 83.1%, and 91.7% of *S. aureus*, respectively. AvBD-12A2 and A3 with a net positive charge of +9 and hydrophobicity of 40% were more effective than AvBD-12A1 (charge = +5, hydrophobicity = 53%). AvBD-12A2 and AvBD-12A3 which had identical charge and hydrophobicity but altered locations of alanine/serine residues exhibited different killing activities against *E. coli* or *P. aeruginosa* (Fig. 3). The bactericidal potency of group-1 analogues can be ranked as AvBD-12A3 > AvBD-12A2 > AvBD-12A1 > AvBD-12. Of the bacterial species tested, *E. coli* and *P. aeruginosa* were more susceptible than *S*. Typhimurium and *S. aureus* to AvBD-12A3 at medium concentrations, ranging from 8 μg/ml to 32 μg/ml (Fig. 3).

**Fig. 4:**
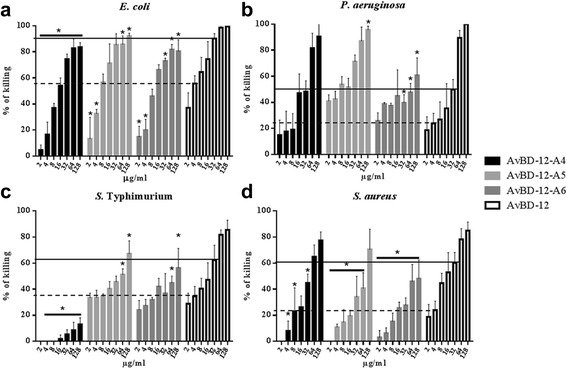
Antimicrobial activity of group 2 analogues. Bacteria (10^5^ CFU/ml) were incubated with peptides at various concentrations, ranging from 2 to 128 μg/ml, at 37 °C for 2 h. Antimicrobial activity was presented as percent of killing compared to non-AvBD treated control. Antimicrobial activity of analogues AvBD-12A4 to A6 against *E. coli* (**a**), *P. aeruginosa* (**b**), *S.* Typhimurium (**c**) and *S. aureus* (**d**). Wild-type AvBD-6 and AvBD-12 were included as controls. Data are means ± SD (*n =* 3). Statistical analysis was performed using the one-way analysis of variance (ANOVA) followed by Duncan’s test for multiple comparisons using software SPSS version 19.0 (IBM Corp., Armonk, NY). Asterisks indicate statistically significant difference between analogues and AvBD-12 at the same concentrations (*p < 0.05). Solid line: average killing percent of 32 μg/ml of wild-type AvBD-12. Dash line: average killing percent of 4 μg/ml of wild-type AvBD-12

**Fig. 5:**
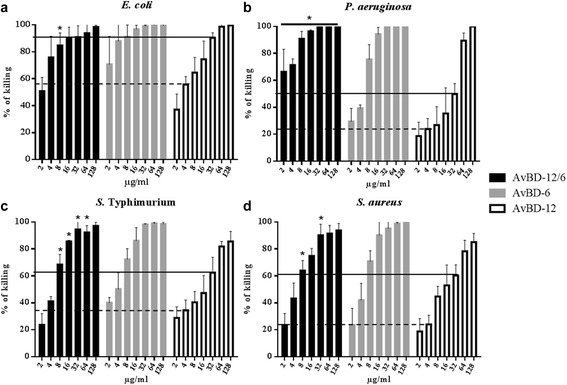
Antimicrobial activity of hybrid peptide AvBD-12/6. Bacteria (10^5^ CFU/ml) were incubated with peptides at various concentrations, ranging from 2 to 128 μg/ml, at 37 °C for 2 h. Antimicrobial activity was presented as percent of killing compared to non-AvBD treated control. Antimicrobial activity of analogue AvBD-12/6 against *E. coli* (**a**), *P. aeruginosa* (**b**), *S.* Typhimurium (**c**) and *S. aureus* (**d**). Wild-type AvBD-6 and AvBD-12 were included as controls. Data are means ± SD (*n =* 3). Statistical analysis was performed using the one-way analysis of variance (ANOVA) followed by Duncan’s test for multiple comparisons using software SPSS version 19.0 (IBM Corp., Armonk, NY). Asterisks indicate statistically significant difference between the hybrid analogue and AvBD-12 at the same concentrations (*p < 0.05). Solid line: average killing percent of 32 μg/ml of wild-type AvBD-12. Dash line: average killing percent of 4 μg/ml of wild-type AvBD-12

Group 2 analogues, including AvBD12A4, AvBD-12A5, and AvBD-12A6 showed similar killing activity to that of AvBD-12 (Fig. 4). One exception was AvBD-12A4 was an exception that lost one disulfide bridge (C^1^-C^5^ or C5-C34) was shown to have weaker action than AvBD-12 against *S*. Typhimurium (*p* < 0.05). Group 3 or the hybrid analogue, AvBD-12/6 with the backbone of AvBD-12 and positively charged amino acid residues of AvBD-6 exhibited similar killing activities to that of AvBD-6 (Fig. 5).

### Minimum inhibitory concentration

The MIC-ls of AvBD-12A1, AvBD-12A2 and AvBD-12A3 against *E. coli*, *S.* Typhimurium, and *P. aeruginosa* were 2 to 16-fold below that of AvBD-12, confirming the improved antimicrobial property of these analogues. The MIC-ls of these analogues against *P. aeruginosa* were 2 to 8-fold below that of AvBD-6. The ratio of MBC-ls/MIC-ls was equal to or below 4:1 for AvBD-12A2 and AvBD-12A3 against the three Gram negative bacterial species tested, suggesting a bactericidal action [29]. The MIC-ls of AvBD-12A4, AvBD-12A5, and AvBD-12A6 with 2, 1 and 0 disulfide bridges, respectively, were higher than that of AvBD-12 and AvBD-6, indicating that removal of disulfide bridges compromised AvBD’s antimicrobial function.

The MIC-ls of AvBD analogues was negatively correlated with the net positive charge. The correlation co-efficiencies (r) for *E. coli*, *S*. Typhimurium, *P. aeruginosa*, and *S. aureus* were −0.7388, *p* < 0.05; −0.8545, *p* < 0.01; −0.8545, *p* < 0.01; and −0.8727, *p* < 0.01, respectively. Although increasing hydrophobicity also resulted in improved antimicrobial potency, a positive correlation between hydrophobicity and antimicrobial activity was not found.

Next, the MICs of AvBD-12A3, the most potent analogue, and the parent AvBD-6 and AvBD-12 were evaluated under conditions outlined in CLSI guidelines [27, 28]. High MICs (128 to 256 μg/ml or above) were obtained (Table 2).

**Table 2:** The minimum inhibitory concentration and minimum bactericidal concentration of AvBD analogues

Bacteria (strain)	*E. coli* (ATCC 25922)			*S.* Typhimurium (ATCC 14028)			*P. aeruginosa* (ATCC 27853)			*S. aureus* (ATCC 29213)		
Defensins	MIC-ls	MBC-ls	MIC	MIC-ls	MBC-ls	MIC	MIC-ls	MBC-ls	MIC	MIC-ls	MBC-ls	MIC
AvBD-12-A1	8	32	N/A	32	128	N/A	32	256	N/A	256	>256	N/A
AvBD-12-A2	4	16	N/A	16	64	N/A	16	32	N/A	256	256	N/A
AvBD-12-A3	4	16	128	16	64	>256	8	16	>256	128	256	>256
AvBD-12-A4	128	>256	N/A	256	>256	N/A	256	>256	N/A	>256	>256	N/A
AvBD-12-A5	64	256	N/A	256	>256	N/A	256	>256	N/A	>256	>256	N/A
AvBD-12-A6	64	256	N/A	256	>256	N/A	256	>256	N/A	>256	>256	N/A
AvBD-12/6	8	32	256	16	64	>256	64	256	>256	256	256	256
AvBD-6	4	16	128	16	64	>256	64	128	>256	256	256	256
AvBD-12	32	128	256	128	256	>256	128	>256	>256	256	256	>256

### Susceptibility to NaCl

**Fig. 6:**
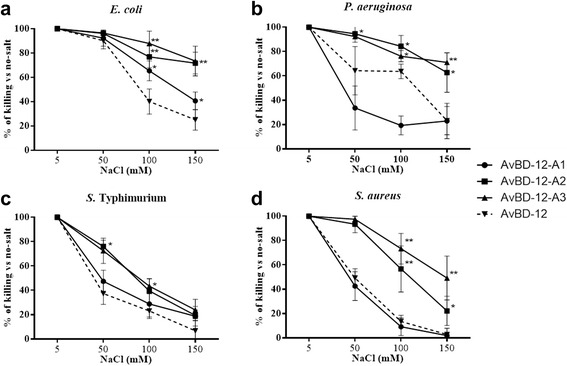
Effect of NaCl on the antimicrobial activity of group one AvBDs. Bacteria were treated with group one analogues in the presence of 5 mM, 50 mM, 100 mM or 150 mM NaCl. AvBDs were used at the following concentrations: 16 μg/ml against *E. coli* (**a**), 32 μg/ml against *P. aeruginosa* (**b**) and *S.* Typhimurium (**c**) and 64 μg/ml against *S. aureus* (**d**). These concentrations were chosen to ensure more than 50% of killing of inoculum by majority of analogues. Results are expressed as percent of killing compared to the no-salt control. Data shown are means ± SD (*n =* 3). Statistical analysis was performed using the one-way analysis of variance (ANOVA) followed by Duncan’s test for multiple comparisons using software SPSS version 19.0 (IBM Corp., Armonk, NY). Asterisks indicate statistically significant difference among different treatment groups (*p < 0.05, **p < 0.01)

**Fig. 7:**
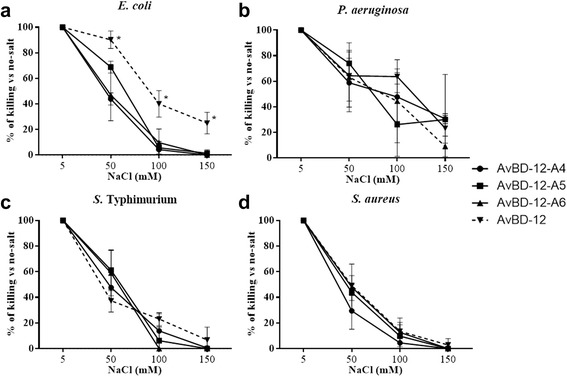
Effect of NaCl on the antimicrobial activity of group two analogues. Bacteria were treated with group two analogues in the presence of 5 mM, 50 mM, 100 mM or 150 mM NaCl. AvBDs were used at the following concentrations: 16 μg/ml against *E. coli* (**a**), 32 μg/ml against *P. aeruginosa* (**b**) and *S*. Typhimurium (**c**) and 64 μg/ml against *S. aureus* (**d**). These concentrations were chosen to ensure more than 50% of killing of inoculum by majority of analogues. Results are expressed as percent of killing compared to the no-salt control. Data shown are means ± SD (*n =* 3). Statistical analysis was performed using the one-way analysis of variance (ANOVA) followed by Duncan’s test for multiple comparisons using software SPSS version 19.0 (IBM Corp., Armonk, NY). Asterisks indicate statistically significant difference among different treatment groups (*p < 0.05, **p < 0.01)

**Fig. 8:**
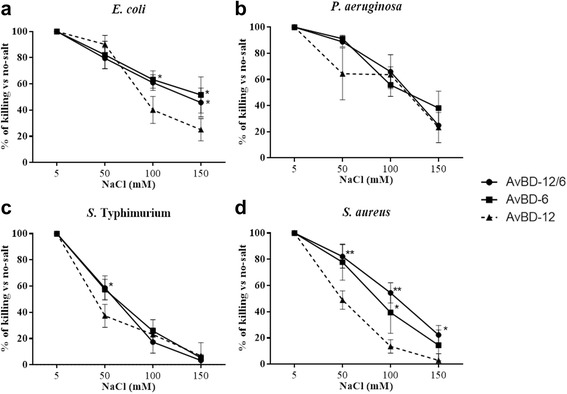
Effect of NaCl on the antimicrobial activity activity of group three analogue AvBD-12/6. Bacteria were treated with AvBDs in the presence of 5 mM, 50 mM, 100 mM or 150 mM NaCl. AvBDs were used at the following concentrations: 16 μg/ml against *E. coli* (**a**), 32 μg/ml against *P. aeruginosa* (**b**) and *S.* Typhimurium (**c**) and 64 μg/ml against* S. aureus* (**d**). ﻿These concentrations were chosen to ensure more than 50% of killing of inoculum by majority of analogues. Results are expressed as percent of killing compared to the no-salt control. Data shown are means ± SD (*n =* 3). Statistical analysis was performed using the one-way analysis of variance (ANOVA) followed by Duncan’s test for multiple comparisons using software SPSS version 19.0 (IBM Corp., Armonk, NY). Asterisks indicate statistically significant difference among different treatment groups (*p < 0.05, **p < 0.01)

The impact of NaCl on the killing activity of AvBDs was assessed at NaCl concentrations ranging from 5 to 150 mM. Increasing NaCl concentration had less adverse impact on the killing activity of AvBD-12A2 and AvBD-12A3 which had a higher net positive charge (+9) and hydrophobicity (40%) than AvBD-12 (Fig. 6). AvBD-12A1, the most hydrophobic (53%) analogue, showed similar or increased susceptibility to NaCl, compared to AvBD-12 (Fig. 6). Susceptibility to NaCl was also influenced by the bacterial species under investigation. At 150 mM NaCl, AvBD-12A2 and AvBD-12A3 retained approximately 80% of killing potency against *E. coli* and *P. aeruginosa*, but only 30% to 60% of killing activity against *S.* Typhimurium and *S. aureus* (Fig. 6). AvBD-12A4, AvBD-12A5, and AvBD-12A6 with fewer disulfide bridges were equally or more susceptible to NaCl than AvBD-12 (Fig. 7). AvBD-12/6 with the backbone of AvBD-12 and increased net positive charge (+5) resembled AvBD-6 instead of AvBD-12 (Fig. 8).

### Cytotoxicity to host cells

Cell cytotoxicity of most potent analogue AvBD-12A3 (+9) and the hybrid analogue AvBD-12/6 (+5) was determined. Exposure of chicken macrophage cell lines HD11 and MQ-NCSU, murine dendritic cell line JAWSII and CHO-K1 cells to AvBDs at concentrations of 4, 16, 64, 256 μg/ml for 4, 12, 24, and 48 h did not affect cell variability. Data on the highest peptide concentration (256 μg/ml) at various exposure times were presented in Additional file 1: Figure S1. The results were consistent with our previous findings that AvBD-6 and AvBD-12 were non-cytotoxic to avian and mammalian cell lines [26].

### Chemotactic activity

**Fig. 9:**
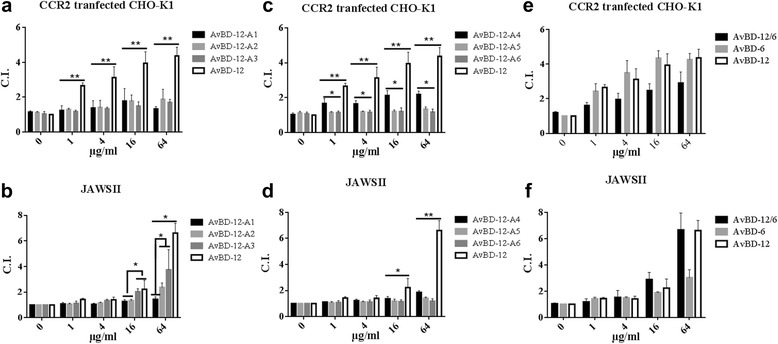
Chemotactic activity of AvBD-12 analogues for CCR2 transfected CHO-K1 cells and mouse immature dendritic JAWSII cells. Migration of CCR2 transfected CHO-K1 cells (**a**-**c**) and mouse immature dendritic JAWSII cells (**d**-**f**). The results are expressed as chemotactic index (C.I.): the number of migrated cells induced by AvBD analogues / the number of migrated cells in response to chemotactic buffer. Data are means ± SD (*n =* 5). Statistical analysis was performed using the one-way analysis of variance (ANOVA) followed by Duncan’s test for multiple comparisons using software SPSS version 19.0 (IBM Corp., Armonk, NY). Asterisks indicate statistically significant difference between analogues and wild-type AvBD-12 (*p < 0.05, **p < 0.01)

Group 1 analogues AvBD-12A1, AvBD-12A2 and AvBS-12A3 showed minimal chemotactic activity for CCR2-CHO cells (Fig. 9a). However, AvBD-12A2 and AvBD-12A3 at 64 μg/ml demonstrated mild (C.I. = 2.37; 35.9% of wild type) and modest (C.I. = 3.74; 56.6% of wild type) chemotactic activity, respectively, for JAWSII cells (Fig. 9b). Analogues AvBD-12A4 with two disulfide bridges had mild chemotactic activity for CCR2-CHO (C.I. = 1.48 to 2.18) and JAWSII cells (C.I. = 1.05 to 1.86) which were significantly below the chemotactic index of parent peptide AvBD-12 (*p* < 0.01, Fig. 9c and d). AvBD-12A5 with one disulfide bridge and AvBD-12A6 with zero disulfide bridges lost their chemotactic activity for both CCR2-CHO-K1 and JAWSII cells (Fig. 9c and d). The hybrid AvBD-12/6 retained the chemotactic function of the backbone peptide AvBD-12 (Fig. 9e and f).

### SEM observation

**Fig. 10:**
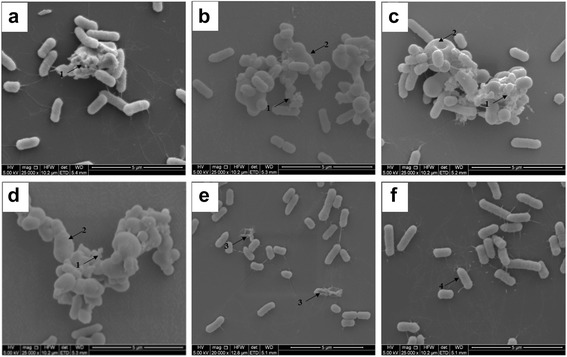
Scanning electron microscopy (SEM) of *S.* Typhimurium treated with AvBDs. Mid-logarithmic-phase *S.* Typhimurium cells (10^8^ CFU) were incubated with AvBDs at a final concentration of 1 × MIC-ls for 30 min. **a** AvBD-12A3, **b** AvBD-12/6, **c** AvBD-12, **d** AvBD-6, **e** Stationary phase bacteria, **f** Mid-logarithmic-phase *S.* Typhimurium treated with PBS. Arrow 1, membrane damage. Arrow 2, cell deformation. Arrow 3, cell death in stationary-phase culture. Arrow 4, a normal cell in mid-logarithmic-phase culture. Scale bar: 5 μm

Mid-logarithmic-phase *S.* Typhimurium bacteria treated with AvBD-12A3 (Fig. 10a), AvBD-12/6 (Fig. 10b), AvBD-12 (Fig. 10c) and AvBD-6 (Fig. 10d) displayed cell membrane damage (arrow 1) and cell deformation (arrow 2). Mid-logarithmic-phase *S.* Typhimurium cells treated with PBS showed normal size and intact structure (Fig. 10f, arrow 4). Cell death in stationary-phase culture (Fig. 10e, arrow 3) showed loss of intracellular content and uniform membrane structure.

## Discussion

Due to the broad spectrum antimicrobial activity, LPS-neutralizing property, immunomodulatory function and the low cell cytotoxicity, defensins may serve as natural antimicrobial peptides or templates for novel drug design [14, 20, 26, 34, 35]. It has been reported that the three conserved disulfide bridges are required for the chemotactic function, but not the antimicrobial activity of mammalian β-defensins [17, 22–25]. However, data from our previous studies indicate that the correct formation of disulfide bridges via oxidative folding is required for maximum antimicrobial activity [21, 26]. In an effort to identify key structural components important to the antimicrobial and chemotactic activities of AvBDs, we compared the predicted three dimensional structures of AvBDs with the well characterized structure of human beta-defensin 6 (hBD6). It was previously shown that the N-terminal α-helix and an adjacent β2-β3 loop form a contiguous binding surface for CCR2 [32]. The presence of a similar CCR2 binding domain in AvBD-12 may account for the chemotactic activity of AvBD-12 for both avian and mammalian immune cells because AvBD-6 which had an N-terminal coil instead of the α-helix was chemotactic only for avian cells [26].

To further understand the structure-activity relationship, we designed and evaluated seven synthetic analogues of AvBD-12. In the first group of analogues, disulfide bridges were removed by replacing cysteines with structurally similar residues (alanine or serine) whereas peptide hydrophobicity and charge were increased by substituting negatively charged residues with hydrophobic (AvBD-12A1) or positively charged residues (AvBD-12A2 and A3). All members of the first group demonstrated stronger antimicrobial activity than their parent peptide AvBD-12, indicating that cysteine-free (linear) AvBDs with a high net positive charge and moderate hydrophobicity can be potent antimicrobial agents. Such linear peptides can be synthesized without oxidative folding which simplifies the production process and reduces cost. In the present study, anti-*S.* Typhimurium activity of AvBD-12A1 decreased significantly as peptide concentration increased from 64 μg/ml to 128 μg/ml (Fig. 3c). Similar results were obtained repeatedly, suggesting that the decrease was unlikely caused by technical errors. We hypothesize that AvBD-12A1, the most hydrophobic (53%) peptide may form aggregates which interferes with peptide binding to *Salmonella* membrane components, resulting in a reduction in antimicrobial potency. Interestingly, AvBD-12A2 and AvBD-12A3 with identical charge and hydrophobicity, but different locations of three alanine and three serine residues (AvBD-12A3: S5S12S17A27A34A35 and AvBD-12A3: A5A12A17S27S34S35) showed significant difference in antimicrobial and chemotactic activities. Structural analysis of AvBD-12A3 revealed an outward protrusion of R29 side chain and parallel twist F28 aromatic ring. We hypothesize that the outward protrusion of a positively charged residue and an adjacent aromatic ring enhances the interaction between AvBD-12A3 and microbial surface components or CCR2, thereby facilitating antimicrobial and chemotactic functions.

To assess the role of disulfide bridges independent of charge and hydrophobicity, we replaced cysteine residues with isosteric α-aminobutyric acids (Abu, U) to create group 2 analogues. Our data indicated that missing even one conserved disulfide bridge resulted in a significant reduction in AvBD’s chemotactic activity. Elimination of two or three disulfide bridges completely abolished the chemotactic activity for both avian CCR2-positive cells and murine immature dendritic cells. It has been shown that C^1^ and C^4^ essential to the formation of first and second disulfide bridges are located at the CCR2 binding center [32]. Thus substitution of C with U may have disrupted the CCR2 binding surface, resulting in the loss of chemotactic activity. Our data also showed that removal of disulfide bridges had varying degrees of negative impact on AvBD’s antimicrobial activity. Analogue AvBD-12A4 missing the C^1^-C^5^ bridge was nearly inactive against *S*. Typhimurium. These results collectively suggest that all three disulfide bridges are needed in the natural form of AvBDs to maintain tertiary structural features critical to interacting with microbial surface components and CCRs on immune cells.

Besides antimicrobial assays, SEM was performed to illustrate the killing mechanisms of AvBD-12 analogues. Treatment of *S*. Typhimurium with AvBD-12A3 and AvBD-12/6, and wild-type AvBD-6 and AvBD-12 caused similar ultrastructural changes, including cell deformation and membrane damage. Giant cells were only observed among treatment groups, indicating membrane permeabilization is an essential step in the killing of microbes by AvBDs.

Salt sensitivity is a major obstacle to the application of β-defensins as chemotherapeutic agents. Different strategies have been explored to increase the resistance of beta-defensins to salts, such as N-terminal deletion [36], combining sequences of HBD-1 and salt-resistant θ-defensin [37] and replacement of tryptophan or histidine with a bulky amino acid β-naphthylalanine [38]. In the present study, we found that increasing peptide charge significantly reduced the impact of NaCl on the antimicrobial efficacy of AvBD-12A2 and AvBD-12A3. However, the activity against S*.* Typhimurium was still severely inhibited by physiological concentration of NaCl (150 mM), suggesting that different mechanisms are involved in killing different bacterial species.

In the present study, we also determined the minimum inhibitory concentrations (MICs) of AvBDs. Using the standard Muller Hinton II broth, the MICs of the parent AvBDs and the most potent analogue AvBD-12A3 were much higher than the MIC values obtained under the low-salt and low-nutrient condition. We hypothesize that interference of initial peptide-bacteria interactions by cationic salts as well as enzymatic degradation or modification of AvBDs during bacterial growth in a nutrient rich Mueller-Hinton broth might have contributed to the decreased antimicrobial activity. It has been shown that human cathelicidin LL-37 could be hydrolyzed by *S. aureus* protease or cleaved by metalloprotease, gelatinase or cysteine protease produced by other bacterial species [39]. Proteolytic degradation is one of the main mechanisms that both gram-positive and gram-negative bacteria use to evade host antimicrobial peptide killing [40–45]. Further modification is clearly needed to improve the efficacy of AvBD-12A3 as an antimicrobial agent. It is noteworthy that all AvBD analogues remain non-cytotoxic to avian and mammalian cells.

## Conclusions

The three conserved disulfide bridges maintaining the tertiary structure of natural AvBDs are required not only for the chemotactic activity, but also for maximum antimicrobial activity. AvBD-12A3 with increased net positive charge and a CCR2-binding domains (N-terminal α-helix and β2-β3 loop) demonstrated potent antimicrobial activity and retained partial chemotactic property. Analogue AvBD-12A3 may serve as a template for the design of novel antimicrobial peptides as therapeutic agents for both avian and mammalian hosts.

## Additional file

Additional file 1: Figure S1. Cytotoxicity of analogues AvBD-12A3 and AvBD-12/6. Effect of 256 μg/ml of AvBD-12A3, AvBD-12/6, AvBD-6 and AvBD-12 on the metabolic activity of MQ-NCSU, HD11, JAWSII and CHO-K1 cells after 4, 12, 24 and 48 hours of incubation. The results are expressed as the percentage of viability relative to the untreated control. The data are means ± SD (*n =* 3). Student *t*-test was performed to analyze differences between AvBD-treated and untreated cells. No significant difference was found. (TIFF 391 kb)

## Abbreviations

(MBC): Minimum bactericidal concentration
ANOVA: One-way analysis of variance
AvBD: Avian beta-defensin
BSA: Bovine serum albumin
C.I.: Chemotaxis index
CFU: Colony-forming unit
DCs: Dendritic cells
ESI-MS: Electrospray ionization mass spectrometry
FBS: Fetal bovine serum
fMLF: N-Formyl-methionyl-leucyl-phenylalanine
Fmoc: 9-fluorenyl-methoxycarbonyl
GFP: Green fluorescent protein
GM-CSF: Granulocyte macrophage colony-stimulating factor
hBD6: Human β-defensin 6, LB: Luria-Bertani
LPS: Lipopolysaccharides
LTA: Lipoteichoic acid
MIC: Minimum inhibitory concentration
PBS: Phosphate buffered saline
RP-HPLC: Reversed-phase high performance liquid chromatography
SD: Standard deviation
SEM: Scanning electron microscopy
TSA: Trypticase soy agar
