# Supplementary figures and images for: Novel synthetic analogues of avian β-defensin-12: the role of charge, hydrophobicity, and disulfide bridges in biological functions
Source: BMC Microbiol. 2017 Feb 23;17:43. doi: 10.1186/s12866-017-0959-9 (PMC5324278; doi:10.1186/s12866-017-0959-9)

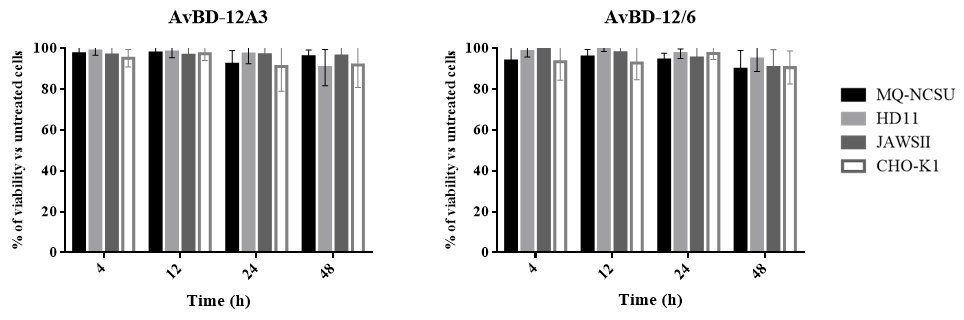

Supplement: Additional file 1: Figure S1. — Cytotoxicity of analogues AvBD-12A3 and AvBD-12/6. Effect of 256 μg/ml of AvBD-12A3, AvBD-12/6, AvBD-6 and AvBD-12 on the metabolic activity of MQ-NCSU, HD11, JAWSII and CHO-K1 cells after 4, 12, 24 and 48 hours of incubation. The results are expressed as the percentage of viability relative to the untreated control. The data are means ± SD (n = 3). Student t-test was performed to analyze differences between AvBD-treated and untreated cells. No significant difference was found. (TIFF 391 kb) [file 12866_2017_959_MOESM1_ESM.tiff]
